# Synthesis and Antitumor Activity of New Thiazole Nortopsentin Analogs

**DOI:** 10.3390/md14120226

**Published:** 2016-12-14

**Authors:** Virginia Spanò, Alessandro Attanzio, Stella Cascioferro, Anna Carbone, Alessandra Montalbano, Paola Barraja, Luisa Tesoriere, Girolamo Cirrincione, Patrizia Diana, Barbara Parrino

**Affiliations:** Dipartimento di Scienze e Tecnologie Biologiche Chimiche e Farmaceutiche, STEBICEF, via Archirafi 32, 90123 Palermo, Italy; virginia.spano@unipa.it (V.S.); alessandro.attanzio@unipa.it (A.A.); stellamaria.cascioferro@unipa.it (S.C.); anna.carbone@unipa.it (A.C.); alessandra.montalbano@unipa.it (A.M.); paola.barraja@unipa.it (P.B.); luisa.tesoriere@unipa.it (L.T.); girolamo.cirrincione@unipa.it (G.C.); patrizia.diana@unipa.it (P.D.)

**Keywords:** marine alkaloids, bis-indolyl alkaloids, thiazolyl-indoles, apoptosis, antiproliferative activity

## Abstract

New thiazole nortopsentin analogs in which one of the two indole units was replaced by a naphthyl and/or 7-azaindolyl portion, were conveniently synthesized. Among these, three derivatives showed good antiproliferative activity, in particular against MCF7 cell line, with GI_50_ values in the micromolar range. Their cytotoxic effect on MCF7 cells was further investigated in order to elucidate their mode of action. Results showed that the three compounds act as pro-apoptotic agents inducing a clear shift of viable cells towards early apoptosis, while not exerting necrotic effects. They also caused cell cycle perturbation with significant decrease in the percentage of cells in the G0/G1 and S phases, accompanied by a concomitant percentage increase of cells in the G2/M phase, and appearance of a subG1-cell population.

## 1. Introduction

**Chart 1:**
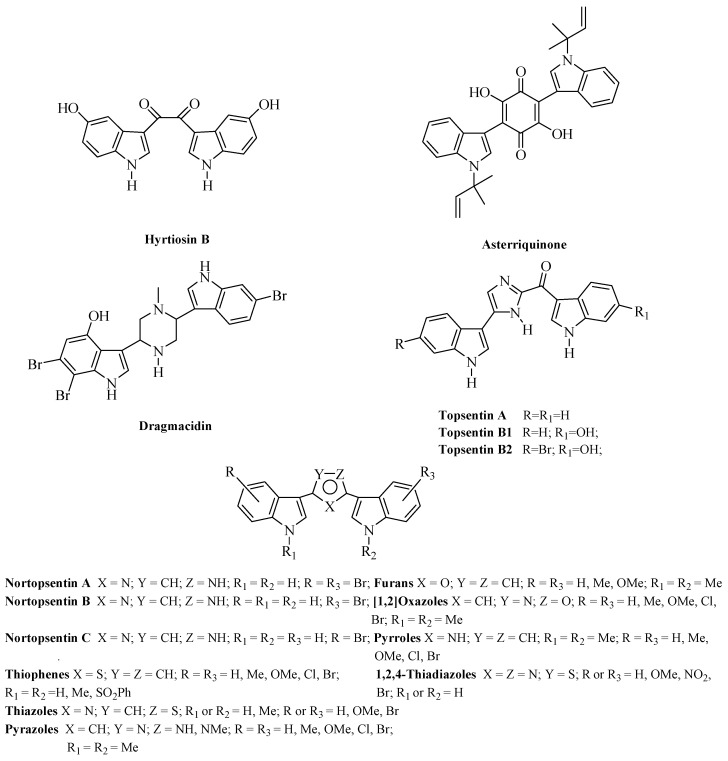
Bis-indolyl alkaloids.

In the latest decades, marine environment has increasingly provided a huge number of biologically active molecules. Among marine organisms, deep-sea sponges have contributed with several compounds endowed with antitumor activity [1,2,3,4]. The isolation of such molecules is very important since cancer is still an important social problem, in fact it is supposed to maintain as causes of death its primacy after heart and circulatory disorders. This scenario justifies the attention paid by a multitude of researchers in the individuation and development of natural or synthetic heterocyclic compounds as scaffold for antitumor agents [5,6,7,8]. Bis-indolyl alkaloids represent an important class of deep-sea sponge metabolites, useful as leads for antitumor agents. They are characterized by two indole units linked, through their position 3, by a spacer [9,10]. The spacer can be an acyclic chain such as in hyrtiosin B, isolated from *Hyrtios erecta* [11], or a carbocyclic ring as in the case of asterriquinone, isolated from *Aspergillus fungi* [12]. Heterocyclic rings can also play as spacer for bis-indolyl alkaloids. Thus, dragmacidin isolated from the deep water sponges *Dragmacidon*, *Halicortex* bears a saturated six-membered piperazine ring (Chart 1) [13].

Topsentins A, B1 and B2, bearing a 2-acyl imidazole spacer, were isolated from *Topsentia genitrix* sponge [14].

Nortopsentins A–C, which exhibit a 2,4-disubstituted imidazole ring as a spacer, were isolated from *Spongosorites ruetzleri*, and showed in vitro cytotoxicity against P388 cells [15].

Due to their interesting cytotoxicity, nortopsentins attracted remarkable attention by researchers and several total syntheses of these natural products were reported [16,17,18,19]. Moreover, the synthesis and antiproliferative evaluation of analogs in which the imidazole ring of the natural compounds was replaced by several five-membered heterocycles such as bis-indolyl-thiophenes [20], -pyrazoles [21], -furans [22], [1,2]oxazoles [22], -pyrroles [23], and -1,2,4-thiadiazoles [24] (Chart 1), many of them showing antiproliferative activity often reaching GI_50_ values in the low micromolar range or even at sub-micromolar level, were reported.

**Chart 2:**
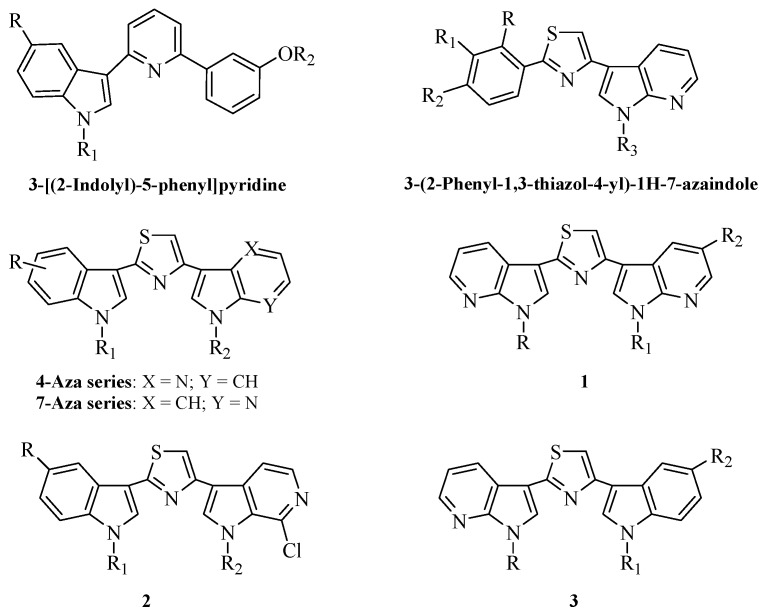
Nortopsentin analogs.

Other structural manipulation of the natural product also involved one or both indole units producing 3-[(2-indolyl)-5-phenyl]pyridine and 3-(2-phenyl-1,3-thiazol-4-yl)-1*H*-7-azaindole derivatives, which showed significant antiproliferative activity and inhibited CDK1 (Chart 2) [25,26].

The interesting results obtained by the aza-substitution of the indole moiety, led to the synthesis and biological evaluation of 3-[2-(1*H*-indol-3-yl)-1,3-thiazol-4-yl]-1*H*-4-azaindoles and the corresponding 1*H*-7-azaindole derivatives (Chart 2) [27,28]. Both series showed potent antiproliferative activity against a wide range of cell lines, including diffuse malignant peritoneal mesothelioma (DMPM), a fatal disease, poorly responsive to conventional therapies, and acted as CDK1 inhibitors. Moreover, a derivative belonging to the 7-aza series, in the mouse model, by intraperitoneal administration was effective in a significant reduction of the DMPM at well tolerated doses.

Lately, three new series of nortopsentin analogs of type **1**, **2** and **3** (Chart 2) were efficiently synthesized and exhibited remarkable antiproliferative activity against several human tumor cell lines [29,30].

Interestingly, a derivative of the series **2** at low concentrations (GI_30_) caused morphological changes typical of autophagic death with massive formation of cytoplasmic acid vacuoles without apparent loss of nuclear material, and with arrest of cell cycle at the G1 phase, whereas higher concentrations (GI_70_) induced apoptosis with arrest of cell cycle at the G1 phase [29].

**Scheme 1:**
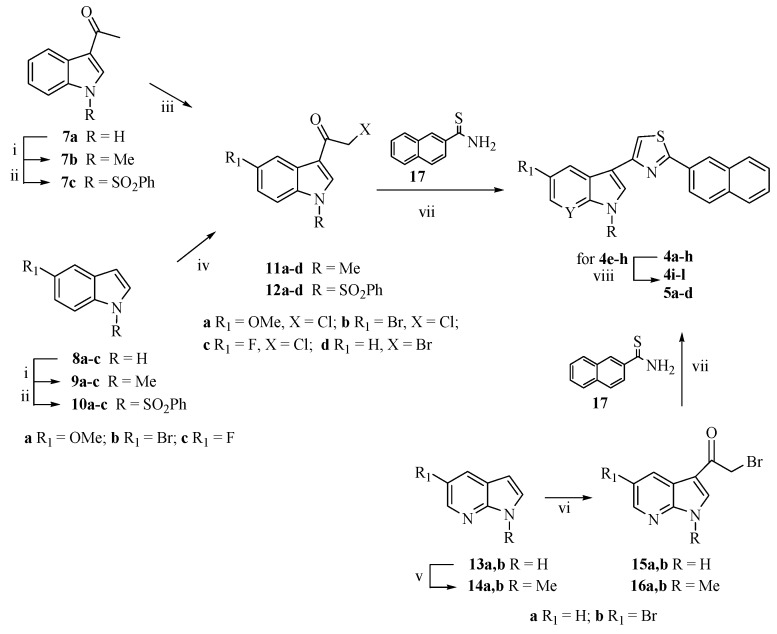
Synthesis of substituted 3-[2-(naphthalen-2-yl)-1,3-thiazol-4-yl]-1*H*-indoles **4** and 3-[2-(naphthalen-2-yl)-1,3-thiazol-4-yl]-1*H*-pyrrolo[2,3-*b*]pyridine **5**. Reagents: (i) (**a**) *t*-BuOK, toluene, TDA-1, rt, 1–24 h; (**b**) MeI, rt, 30 min–2 h, 80%–98%; (ii) (**a**) NaH, THF, 0 °C-rt, 1 h; (**b**) benzensulphonyl chloride, rt, 1–24 h, 87%–90%; (iii) Br_2_, methanol, reflux, 2 h, 40%–70%; (iv) AlCl_3_, DCM, ClCOCH_2_Cl, rt, 1–5 h, 60%–85%; (v) (**a**) *t-*BuOK, toluene, TDA-1, rt, 3 h; (**b**) MeI, rt, 1 h, 96%–99%; (vi) AlCl_3_, DCM, BrCOCH_2_Br, reflux, 40 min, 70%–92%; (vii) ethanol, 60 °C-reflux, 30 min–12 h, 48%–95%; and (viii) NaOH, water, ethanol, reflux, 5–6 h, 50%–80%.

**Scheme 2:**
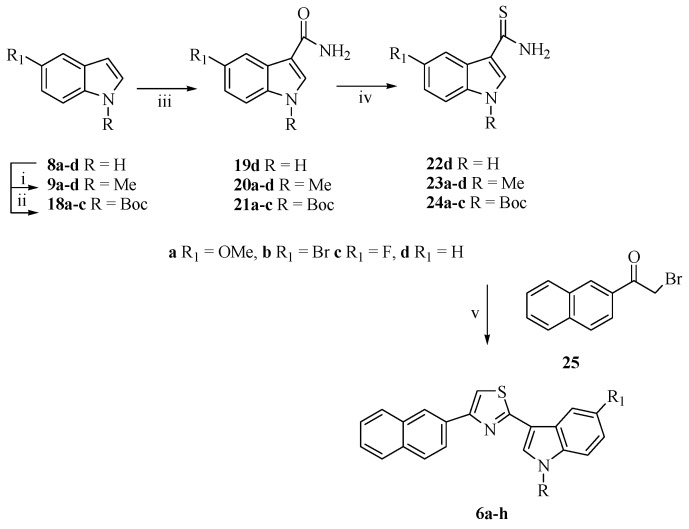
Synthesis of 3-[4-(naphthalene-2-yl)-1,3-thiazol-2-yl]-1*H*-indoles **6a**–**h**. Reagents: (i) (**a**) *t-*BuOK, toluene, TDA-1, rt, 6 h; (**b**) MeI, rt, 1 h, 96%–98%; (ii) Boc, triethylamine, THF, reflux, 24–48 h, 90%–100%; (iii) (**a**) chlorosulphonyl isocyanide, acetonitrile, 0 °C then rt 0.5–2 h or reflux, 15 min; (**b**) acetone/water 8:1, KOH 10%, 40%–70%; (iv) Lawesson’s reagent, toluene, reflux, 0.5–24 h, 90%–98%; (v) DMF, 60 °C-reflux, 3–24 h, 48%–99%.

Considering the interesting biological activity of nortopsentin analogs and in particular of 3-(2-phenyl-1,3-thiazol-4-yl)-1*H*-7-azaindole derivatives previously reported by us [26], herein we report the synthesis of new derivatives of type **4**, **5** (Scheme 1) and **6** (Scheme 2), in which one of the two indole units was replaced by a naphthyl portion, to further investigate the contribution of the aryl moiety on biological activity. The antiproliferative activity of the novel compounds was evaluated in different human cancer cell lines and further studies were performed on the most active derivatives, in order to clarify their mechanism of action.

## 2. Results and Discussion

### 2.1. Chemistry

3-[2-(Naphthalen-2-yl)-1,3-thiazol-4-yl]-1*H*-indoles of type **4** and 3-[2-(naphthalen-2-yl)-1,3-thiazol-4-yl]-1*H*-pyrrolo[2,3-*b*]pyridines of type **5** (Table 1) were conveniently synthesized by Hantzsch reaction between naphthalene-2-carbothioamide **17** and 3-haloacetyl compounds of type **11**, **12**, **15** and **16** (Scheme 1).

3-Haloacetyl intermediates **11c**, **15a**,**b** and **16a**,**b** were obtained from the corresponding indole **9c** or 7-azaindoles **13a**,**b** and **14a**,**b** respectively, while compounds **11d** was synthesized from the corresponding *N*-methyl-1-(1*H*-indol-3-yl)ethanone **7b**, as previously reported [29,30].

3-Haloacetyl compounds **11a**,**b** and **12a**–**c** were prepared (70%–85% and 60%–70%, respectively), reacting their corresponding *N*-methyl or *N*-SO_2_Ph derivatives **9a**,**b** and **10a**–**c** with chloroacetyl chloride (ClCOCH_2_Cl) in presence of aluminum chloride (AlCl_3_) in dichloromethane (DCM); compound **12d** was obtained (70%) from the corresponding *N*-SO_2_Ph 1-(1*H*-indol-3-yl)ethanone **7c** using bromine in refluxing methanol (MeOH).

Reaction of the synthesized key intermediates **11a**–**d**, **12a**–**d**, **15a**,**b** and **16a**,**b** with naphthalene-2-carbothioamide **17** in refluxing ethanol gave the 3-[2-(naphthalen-2-yl)-1,3-thiazol-4-yl]-1*H*-indoles **4a**–**h** (48%–95%) and 3-[2-(naphthalen-2-yl)-1,3-thiazol-4-yl]-1*H*-pyrrolo[2,3-*b*]pyridine **5a**–**d** (55%–85%), respectively.

*N-*SO_2_Ph protected indoles **10a**–**c** [31,32] and 1-(1*H*-indol-3-yl)ethanone **7c** (90%) were synthesized from the commercially available indoles **8a**–**c** or 1-(1*H*-indol-3-yl)ethanone **7a** by reaction with benzenesulphonyl chloride and sodium hydride (NaH), in tetrahydrofuran (THF); whereas methylated compounds **9a**–**c** and **7b** were prepared as previously reported [26,30].

The subsequent deprotection of *N*-SO_2_Ph derivatives **1e**–**h** using sodium hydroxide in ethanol under reflux afforded, after neutralization, the corresponding unprotected derivatives **4i**–**l** (50%–80%).

3-[4-(Naphthalene-2-yl)-1,3-thiazol-2-yl]-1*H*-indoles **6a**–**h** were synthesized (Table 2), also in this case, by Hantzsch reaction between the key intermediates indolo-3-carbothiamides **22d**, **23a**–**d**, **24a**–**c** and naphthalene-2-acetylbromide **25**, performed in dimethylformamide (DMF) under reflux (Scheme 2). In particular, reaction of naphthalene-2-acetylbromide **25** with *N*-Boc indolo-3-carbothiamides **24a**–**c** afforded the corresponding unprotected 3-[4-(naphthalene-2-yl)-1,3-thiazol-2-yl]-1*H*-indoles **6e**–**h**. Indolo-3-carbothiamides **22d**, **23a**–**d** and **24a**–**c** were prepared from the corresponding indoles **8a**–**d**, **9a**–**d** and **18a**–**c** through the formation of amides **19d**, **20a**–**d** and **21a**–**c** as previously reported by us [28].

### 2.2. Biology

#### 2.2.1. Cytotoxic Activity

All synthesized nortopsentin analogs 3-[2-(naphthalen-2-yl)-1,3-thiazol-4-yl]-1*H*-indoles **4a**–**l** 3-[2-(naphthalen-2-yl)-1,3-thiazol-4-yl]-1*H*-pyrrolo[2,3-*b*]pyridines **5a**–**d**, and 3-[4-(naphthalene-2-yl)-1,3-thiazol-2-yl]-1*H*-indoles **6a**–**h**, were tested at a single dose (10^−5^ M) for cytotoxicity against three human tumor cell lines, HCT 116 cells (colorectal carcinoma), MDA-MB-435 cells (melanoma) and MCF-7 cells (breast cancer) by MTT assay. In Table 3 are shown the growth percentages calculated for some of the nortopsentin analogs since those derivatives for which growth percentages higher than 90 were measured against all the three lines are not reported. 

**Figure 1:**
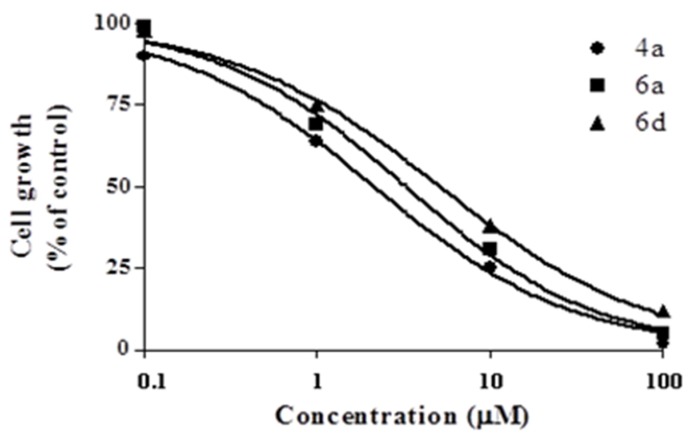
Effect of compounds **4a**, **6a** and **6d** on the growth of MCF-7. Cells were treated with the compounds for 72 h and cell survival was measured by MTT assay in comparison to cells treated with vehicle alone (control), as reported in Section 3.2. Values are the mean ± SD of three separate experiments carried out in triplicate.

Compounds **4a**, **6a** and **6d** appeared the most active compounds in inhibiting cell growth and their activity was further investigated on MCF-7 cells, which are the most sensitive to the cytotoxic property of the compounds. When assayed in the concentration range 0.1–100 µM, they inhibited the growth of MCF-7 cells in dose-dependent manner (Figure 1) and on the basis of GI_50_ value, the drug concentration effective in causing 50% inhibition of cell growth, compound **4a** appeared the most effective (Table 4).

#### 2.2.2. Cell Death Mechanism

**Figure 2:**
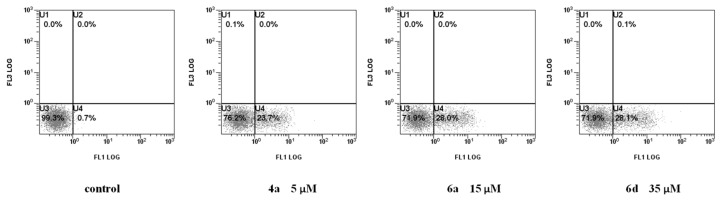
Flow cytometric analysis for the quantification by Annexin V/PI double staining of compounds **4a**, **6a** and **6d** induced apoptosis in MCF-7 cells. Cell monolayers were incubated in the absence (control) or in the presence of the compounds at their relevant GI_50_ values. After 24 h incubation, cells were submitted to double staining with Annexin V/PI as reported in Section 3.2. U3, viable cells (Annexin V−/PI−); U4, cells in early apoptosis (Annexin V+/PI−); U2, cells in tardive apoptosis (Annexin V+/PI+); U1, necrotic cells (Annexin V−/PI+). Representative images of three experiments with comparable results.

The mechanism of the most active compounds, **4a**, **6a** and **6d**, in inducing cell death (necrosis or apoptosis) was investigated by double staining with propidium iodide (PI) and Annexin V-FITC followed by cytofluorimetric analysis. As shown in Figure 2, all three compounds induced a clear shift of viable cells towards early apoptosis in MCF-7 cells after 24 h treatment, while did not exert necrotic effects.

#### 2.2.3. Cell Cycle Analysis

**Figure 3:**
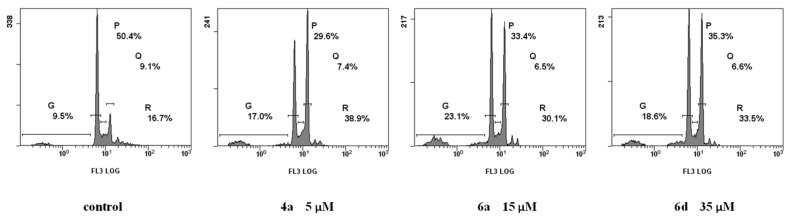
Cell cycle analysis of MCF-7 cells treated with compounds **4a**, **6a** and **6d**. Cell monolayers were incubated in the absence (control) or in the presence of the compounds at their relevant GI_50_ values. After 24 h incubation, propidium iodide-stained cells were submitted to flow cytometric analysis as reported in Section 3.2. Representative images of three experiments with comparable results.

The distribution of MCF-7 cells in the cell cycle phases after 24 h treatment with the three compounds **4a**, **6a** and **6d**, was assessed by flow cytometric analysis after staining of DNA with PI. All synthesized compounds caused a significant decrease in the percentage of cells in the G0/G1 and S phases, accompanied by a concomitant percentage increase of cells in the G2/M phase, and appearance of a subG1-cell population (Figure 3).

## 3. Materials and Methods

### 3.1. Chemistry

#### 3.1.1. General

All melting points were taken on a Büchi-Tottoly capillary apparatus. IR spectra were determined in bromoform with a Shimadzu FT/IR 8400S spectrophotometer. ^1^H and ^13^C NMR spectra were measured at 200 and 50.0 MHz, respectively, in dimethylsulfoxide (DMSO)-*d*_6_ solution, using a Bruker Avance II series 200 MHz spectrometer. Compounds **5c**,**d** were characterized only by ^1^H NMR spectra because of their poor solubility. Column chromatography was performed with Merk silica gel 230–400 mesh ASTM or with Büchi Sepacor chromatography module (prepacked cartridge system). Elemental analyses (C, H, N) were within ±0.4% of theoretical values and were performed with a VARIO EL III elemental analyzer. Purity of all the tested compounds was greater than 98%, determined by HPLC. Compounds **7b** [30], **9a**–**d** [26], **10a**–**c** [31,32], **11c**,**d** [30], **14a**,**b**, **15a**,**b**, **16a**,**b**, [29] **18a**–**c**, **20a**–**d**, **21a**–**c**, **23a**–**d** and **24a**–**c** [28] were prepared as previously described by us.

#### 3.1.2. Synthesis of 1-[1-(Phenylsulfonyl)-1*H*-indol-3-yl]ethanone (**7c**)

To a solution of the 3-acetylindole **7a** (12.6 mmol) in anhydrous THF (15.0 mL) sodium hydride (60% dispersion in mineral oil, 0.6 g, 12.6 mmol) was added at 0 °C and the mixture was stirred at room temperature for 1 h. Benzensulfonyl chloride (1.6 mL, 12.6 mmol) was added and the mixture was stirred at room temperature for 1–24 h. The residue was evaporated under reduced pressure, treated with water (50 mL) and extracted with EtOAc (3 × 50 mL). The organic phase was dried (Na_2_SO_4_), evaporated under reduced pressure and purified by column chromatography using DCM as eluent. Yield 90%; analytical and spectroscopic data were previously reported [33].

#### 3.1.3. Synthesis of Substituted 2-Chloro-1-(1-methyl-1*H*-indol-3-yl)ethanones (**11a**,**b**) and 2-Chloro-1-[1-(phenylsulfonyl)-1*H*-indol-3-yl]ethanones (**12a**–**c**)

A solution of the suitable indole **9a**,**b**, **10a**–**c** (3.1 mmol) in anhydrous DCM (12.0 mL) was added dropwise at 0 °C, under nitrogen atmosphere, to a stirred suspension of aluminum chloride (2.9 g, 21.7 mmol) in anhydrous DCM (46.0 mL). Then, chloroacetyl chloride (0.8 mL, 9.3 mmol) was slowly added to the reaction mixture, which was stirred at room temperature for 1–5 h and then poured in ice and water (60 mL) and extracted with DCM (3 × 60 mL). The organic phase was dried (Na_2_SO_4_), evaporated under reduced pressure and purified by column chromatography using DCM as eluent.

##### 2-Chloro-1-(5-methoxy-1-methyl-1*H*-indol-3-yl)ethanone (**11a**)

Conditions: room temperature for 1 h; white solid; yield 85%; mp: 227–228 °C; IR: 1653 (CO) cm^−1^; ^1^H NMR (200 MHz, DMSO-*d*_6_) δ: 3.80 (s, 3H, CH_3_), 3.85 (s, 3H, CH_3_), 4.80 (s, 2H, CH_2_), 6.94 (dd, 1H, *J* = 2.5, 8.9 Hz, H-6), 7.49 (d, 1H, *J* = 8.9 Hz, H-7), 7.68 (d, 1H, *J* = 2.5 Hz, H-4), 8.40 (s, 1H, H-2); ^13^C NMR (50 MHz, DMSO-*d*_6_) δ: 33.4 (q), 45.9 (t), 55.1 (q), 102.8 (d), 111.6 (d), 112.1 (s), 112.8 (d), 126.5 (s), 132.0 (s), 138.2 (d), 155.9 (s), 185.3 (s). Anal. Calcd. for C_12_H_12_ClNO_2_ C (60.64%) H (5.09%) N (5.89%) found C (60.32%) H (5.12%) N (5.75%).

##### 1-(5-Bromo-1-methyl-1*H*-indol-3-yl)-2-chloroethanone (**11b**)

Conditions: room temperature for 5 h; dark brown solid; yield 70%; mp: 175–176 °C; IR 1658 (CO); ^1^H NMR (200 MHz, DMSO-*d*_6_) δ: 3.89 (s, 3H, CH_3_), 4.85 (s, 2H, CH_2_), 7.46 (dd, *J* = 2.0, 8.7 Hz, 1H, H-6), 7.59 (d, *J* = 8.7 Hz, 1H, H-7), 8.30 (d, *J* = 2.0 Hz, 1H, H-4), 8.51 (s, 1H, H-2); ^13^C NMR (50 MHz, DMSO) δ: 33.6 (q), 46.2 (t), 111.8 (s), 113.2 (d), 115.4 (s), 123.3 (d), 125.8 (d), 127.4 (s), 136.1 (s), 139.3 (d), 186.8 (s). Anal. Calcd. for C_11_H_9_BrClNO C (46.11%) H (3.17%) N (4.89%) found C (46.28%) H (3.54%) N (5.01%).

##### 2-Chloro-1-[5-methoxy-1-(phenylsulfonyl)-1*H*-indol-3-yl]ethanone (**12a**)

Conditions: room temperature for 1 h; dark brown solid; yield 70%; mp: 168–169 °C; IR: 1683 (CO), 1448, 1477 (SO_2_) cm^−1^; ^1^H NMR (200 MHz, DMSO) δ: 3.79 (s, 3H, CH_3_), 5.15 (s, 2H, CH_2_), 7.05 (dd, 1H, *J* = 2.6, 9.1 Hz, H-6), 7.82–7.58 (m, 4H, ArH), 7.88 (d, 1H, *J* = 9.1 Hz, H-7), 8.20–8.05 (m, 2H, ArH), 8.92 (s, 1H, H-2); ^13^C NMR (50 MHz, DMSO-*d*_6_) δ: 47.1 (t), 55.4 (q), 104.2 (d), 114.0 (d), 115.0 (d), 117.6 (s), 127.1 (dx2), 128.1 (s), 128.4 (s), 130.1 (dx2), 134.9 (d), 135.3 (d), 136.3 (s), 157.2 (s), 187.4 (s). Anal. Calcd. for C_17_H_14_ClNO_4_S C (56.12%) H (3.88%) N (3.85%) found C (55.91%) H (3.98%) N (4.07%).

##### 1-[5-Bromo-1-(phenylsulfonyl)-1*H*-indol-3-yl]-2-chloroethanone (**12b**)

Conditions: room temperature for 1 h; brown solid; yield 70%; mp: 168–169 °C; IR: 1689 (CO), 1366, 1442 (SO_2_) cm^−1^; ^1^H NMR (200 MHz, DMSO-*d*_6_) δ: 5.20 (s, 2H, CH_2_), 7.63–7.75 (m, 4H, H-6, ArH), 8.00 (d, 1H, *J* = 8.9 Hz, H-7), 8.17–8.21 (m, 2H, H-4, ArH), 8.32 (d, 1H, *J* = 1.8 Hz, ArH), 9.07 (s, 1H, H-2); ^13^C NMR (50 MHz, DMSO-*d*_6_) δ: 47.1 (t), 115.1 (d), 116.9 (s), 118.0 (s),124.3 (s), 127.2 (dx2), 128.7 (d), 128.8 (d), 130.2 (dx2), 132.8 (s), 134.5 (d), 135.6 (d), 136.0 (s), 187.3 (s). Anal. Calcd. for C_16_H_11_BrClNO_3_S C (46.57%) H (2.69%) N (3.39%) found C (46.35%) H (2.87%) N (3.25%).

##### 2-Chloro-1-[5-fluoro-1-(phenylsulfonyl)-1*H*-indol-3-yl]ethanone (**12c**)

Conditions: room temperature for 1 h; light brown; yield 60%; mp: 126–127 °C; IR: 1688 (CO), 1376, 1447 (SO_2_) cm^−1^; ^1^H NMR (200 MHz, DMSO-*d*_6_) δ: 5.16 (s, 2H, CH_2_), 7.33 (td, 1H, *J* = 2.7, 9.2 Hz, H-6), 7.66 (m, 3H, ArH, H-7), 7.86 (dd, 1H, *J* = 9.2, 2.6 Hz, ArH), 8.02 (dd, 1H, *J* = 9.2, 4.3 Hz, ArH), 8.16 (m, 2H, ArH), 9.04 (s, 1H, H-2); ^13^C NMR (50 MHz, DMSO-*d*_6_) δ: 47.1 (t), 107.6 (d, *J_C4-F_* = 25.1 Hz), 113.9 (d), 114.8 (d, *J_C7-F_* = 9.6 Hz), 114.9 (d), 117.4 (s), 117.5 (s), 127.2 (dx2), 130.2 (dx2), 130.4 (s), 135.8 (d, *J_C6-F_* = 21.9 Hz), 136.1 (s), 159.2 (s, *J_C5-F_* = 299.5 Hz) 187.3 (s); Anal. Calcd. for C_16_H_11_ClFNO_3_S C (54.63%) H (3.15%) N (3.98%) found C (54.38%) H (2.87%) N (4.22%).

#### 3.1.4. Synthesis of 3-(1-Benzenesulfonyl-1*H*-indol-3-yl)-2-bromoethanone (**12d**)

To a stirred solution of 1-[1-(phenylsulfonyl)-1H-indol-3-yl]ethanone **7c** (0.5 g, 1.7 mmol) in ethanol (15.0 mL), bromine (0.1 mL, 2 mmol) was added dropwise under nitrogen atmosphere. The reaction mixture was heated under reflux for 2 h. After cooling the solvent was evaporated under reduced pressure. The residue was treated with water (20 mL), made alkaline by adding sodium hydrogen carbonate (150 mg) and extracted with EtOAc (3 × 50 mL). The organic phase was dried (Na_2_SO_4_), evaporated under reduced pressure and purified by column chromatography using cycloexane/ethyl acetate 95:5 as eluent. Yield 70%; analytical and spectroscopic data were in accordance with those previously reported [34].

#### 3.1.5. Synthesis of 5-Substituted-3-[2-(naphthalen-2-yl)-1,3-thiazol-4-yl]-1-(protected)-1*H*-indoles (**4a**–**h**)

A suspension of the proper 3-haloacetyl derivative **11a**–**d** or **12a**–**d** (0.84 mmol) and naphthalene-2-carbothioamide **17** (0.16 g, 0.84 mmol), in anhydrous ethanol (5.0 mL), was heated under reflux for 30 min–6 h or at 60 °C for 12 h. The solid formed was filtered, dried, an purified by column chromatography using cycloexane/ethyl acetate as eluent.

##### 5-Methoxy-1-methyl-3-[2-(naphthalen-2-yl)-1,3-thiazol-4-yl]-1*H*-indole (**4a**)

Conditions: reflux for 1 h; cycloexane/ethyl acetate 7:3; light yellow solid; yield 95%; mp: 168–169 °C; ^1^H NMR (200 MHz, DMSO-*d*_6_) δ: 3.85 (s, 3H, CH_3_), 3.90 (s, 3H, CH_3_), 6.99 (dd, 1H, *J =* 2.4, 8.9 Hz, H-6″), 7.42 (d, 1H, *J* = 8.9 Hz, H-7″), 7.58–7.63 (m, 1H, ArH), 7.72 (d, 1H, *J* = 2.4 Hz, H-4″), 7.88 (s, 1H, H-2″), 7.97–8.02 (m, 3H, ArH), 8.06–8.13 (m, 2H, ArH), 8.21 (dd, 1H, *J* = 1.7, 8.6 Hz, ArH), 8.61 (s, 1H, H-5); ^13^C NMR (50 MHz, DMSO-*d*_6_) δ: 32.7 (q), 55.4 (q), 120.2 (d), 109.5 (s), 190.9 (d), 110.0 (s), 111.0 (d), 111.6 (d), 123.6 (d), 125.3 (d), 127.0 (d), 127.1 (d), 127.8 (d), 128.5 (d), 128.9 (d), 129.7 (d), 130.7 (s), 130.3 (s), 132.9 (s), 133.6 (s), 151.7 (s), 154.3 (s), 165.8 (s). Anal. Calcd. for C_23_H_18_N_2_OS C=C (74.57%) H (4.90%) N (7.56%) found C (74.85%) H (4.63%) N (7.73%).

##### 5-Bromo-1-methyl-3-[2-(naphthalen-2-yl)-1,3-thiazol-4-yl]-1*H*-indole (**4b**)

Conditions: reflux for 1 h; cycloexane/ethyl acetate 7:3; light brown solid; yield 72%; mp: 124–125 °C; ^1^H NMR (200 MHz, DMSO-*d*_6_) δ: 3.90 (s, 3H, CH_3_), 7.40 (dd, 1H, *J =* 1.8, 8.7 Hz, H-6″), 7.50 (d, 1H, *J =* 8.7, H-7″), 7.60 (m, 2H, ArH), 7.97–8.03 (m, 2H, H-4″, H-2″), 8.08–8.12 (m, 3H, ArH), 8.20 (dd, 1H, *J* = 1.8, 9.6 Hz, ArH), 8.40 (d, 1H, *J =* 1.8 Hz; ArH), 8.60 (s, 1H, H-5); ^13^C NMR (50 MHz, DMSO-*d*_6_) δ: 32.9 (q), 109.6 (s), 111.0 (d), 112.4 (d), 112.9 (s), 112.5 (d), 116.4 (s), 123.5 (d), 124.2 (d), 125.4 (d), 126.5 (s), 127.0 (d), 127.2 (d), 127.8 (d), 128.5 (d), 128.9 (d), 130.6 (d), 132.9 (s), 133.6 (s), 135.7 (s), 150.8 (s), 166.2 (s). Anal. Calcd. for C_22_H_15_BrN_2_S C (63.01%) H (3.61%) N (6.68%) found C (62.89%) H (3.85%) N (6.44%).

##### 5-Fluoro-1-methyl-3-[2-(naphthalen-2-yl)-1,3-thiazol-4-yl]-1*H*-indole (**4c**)

Conditions: 60 °C for 12 h; cycloexane/ethyl acetate 7:3; brown solid; yield 48%; mp: 151–152 °C; ^1^H NMR (200 MHz, DMSO-*d*_6_) δ: 3.90 (s, 3H, CH_3_), 7.15 (td, 1H, *J* = 2.4, 9.1 Hz, H-6″), 7.53–7.65 (m, 3H, ArH, H-7″, H-4″), 7.93 (s, 1H, H-2″), 7.98–8.04 (m, 2H, ArH), 8.07–8.15 (m, 3H, ArH), 8.22 (dd, 1H, *J* = 1.6, 8.6 Hz, ArH ), 8.61 (s, 1H, H-5); ^13^C NMR (50 MHz, DMSO-*d*_6_) δ: 32.9 (q), 105.2 (d, *J_C-4″-F_* = 23.9 Hz), 109.6 (d), 110.1 (d), 110.4 (d), 111.4 (d), 111.6 (d), 123.6 (d), 125.0 (s), 125.1 (s), 125.3 (d), 127.0 (d), 127.5 (d *J_C-6″-F_* = 29.4 Hz), 128.7 (d, *J_C-7″-F_* = 16.3 Hz), 131.0 (d), 130.6 (s), 132.9 (s), 133.6 (s), 133.8 (s), 151.1 (s), 166.0 (s). Anal. Calcd. for C_22_H_15_FN_2_S C (73.72%) H (4.22%) N (7.82%) found C (73.98%) H (4.56%) N (7.631%).

##### 1-Methyl-3-[2-(naphthalen-2-yl)-1,3-thiazol-4-yl]-1*H*-indole (**4d**)

Conditions: reflux for 1 h; cycloexane/ethyl acetate 7:3; brown solid; yield 75%; mp: 174–175 °C; ^1^H NMR (200 MHz, DMSO-*d*_6_) δ: 3.90 (s, 3H, CH_3_), 7.19–7.32 (m, 2H, H-6″, H-7″), 7.52–7.65 (m, 2H, H-4″, ArH), 7.88 (s, 1H, H-2″), 7.98–8.15 (m, 4H, ArH), 8.20–8.28 (m, 2H, ArH), 8.61 (s, 1H, H-5); ^13^C NMR (50 MHz, DMSO-*d*_6_) δ: 109.9 (s), 110.2 (d), 110.3 (d), 120.0 (d), 120.3 (d), 121.7(d), 124.9 (s), 125.3 (d), 126.7 (d), 127.0 (d), 127.1 (d), 127.8 (d), 128.6 (d), 128.9 (d), 129.2 (d), 130.7 (s), 132.9 (s), 133.6 (s), 137.0 (s), 151.6 (s), 165.9 (s). Anal. Calcd. for C_22_H_16_N_2_S C (77.62%) H (4.74%) N (8.23%) found C (77.45%) H (4.79%) N (7.98%).

##### 5-Methoxy-3-[2-(naphthalen-2-yl)-1,3-thiazol-4-yl]-1-(phenylsulfonyl)-1*H*-indole (**4e**)

Conditions: reflux for 1 h; cycloexane/ethyl acetate 9:1; white solid; yield 90%; mp: 168–169 °C; IR: 1451, 1526 (SO_2_) cm^−1^; ^1^H NMR (200 MHz, DMSO-*d*_6_) δ: 3.89 (s, 3H, CH_3_), 7.06 (dd, 1H, *J =* 2.5, 9.0 Hz, H-6″), 7.56–7.75 (m, 5H, H-7″, ArH), 7.82 (d; 1H, *J =* 2.5 Hz, H-4″), 7.92–8.14 (m, 6H, ArH), 8.23 (dd, 1H, *J =* 1.7, 8.6 Hz, ArH), 8.33 (s, 1H, H-2″), 8.44 (s, 1H, H-5), 8.64 (m, 1H, ArH); ^13^C NMR (50 MHz, DMSO-*d*_6_) δ: 55.43 (q), 104.3 (d), 110.3 (s), 114.1 (d), 114.2 (d), 115.5 (d), 117.6 (s), 123.5 (d), 125.5 (d), 125.6 (d), 126.7 (d), 127.1 (dx2), 127.3 (d), 127.8 (d), 128.6 (d), 128.9 (d), 129.2 (s), 129.9 (dx2), 130.3 (s), 132.9 (s), 133.7 (s), 134.7 (d), 136.7 (s), 148.6 (s), 156.5 (s), 166.8 (s). Anal. Calcd. for C_28_H_20_N_2_O_3_S_2_ C (67.72%) H (4.06%) N (5.64%) found C (67.55%) H (4.23%) N (5.77%).

##### 5-Bromo-3-[2-(naphthalen-2-yl)-1,3-thiazol-4-yl]-1-(phenylsulfonyl)-1*H*-indole (**4f**)

Conditions: reflux for 6 h; cycloexane/ethyl acetate 8:2; white solid; yield 70%; mp: 216–217 °C; IR: 1451, 1526 (SO_2_) cm^−1^; ^1^H NMR (200 MHz, DMSO-*d*_6_) δ: 7.57–7.78 (m, 6H, ArH), 7.96–8.04 (m, 2H, ArH), 8.08–8.14 (m, 4H, ArH), 8.20 (dd, 1H, *J =* 1.8, 8.1 Hz, ArH), 8.38 (s, 1H, H-2″), 8.52 (d, 1H, *J =* 1.8, ArH), 8.56 (s, 1H, H-5), 8.62 (s, 1H, ArH); ^13^C NMR (50 MHz, DMSO-*d*_6_) δ: 115.3 (d),116.1 (d), 116.8 (s), 117.0 (s), 123.5 (d), 124.2 (d), 125.7 (d), 126.1 (d), 126.8 (d), 127.1 (dx2), 127.4 (d), 127.8 (d), 128.0 (d), 128.6 (d), 129.0 (d), 129.6 (s), 130.0 (dx2), 130.2 (s), 132.9 (s), 133.5 (s), 133.7 (s), 135.0 (d), 136.5 (s), 147.9 (s), 167.1 (s). Anal. Calcd. for C_27_H_17_BrN_2_O_2_S_2_ C (59.45%) H (3.14%) N (5.14%) found C (59.69%) H (3.34%) N (5.38%).

##### 5-Fluoro-3-[4-(naphthalen-2-yl)-1,3-thiazol-2-yl]-1-(phenylsulfonyl)-1*H*-indole (**4g**)

Conditions: reflux for 2 h; cycloexane/ethyl acetate 8:2; white solid; yield 60%; mp: 193–194 °C; IR: 1447, 1375 (SO_2_) cm^−1^; ^1^H NMR (200 MHz, DMSO-*d*_6_) δ: 7.34 (td, *J =* 2.5, 9.1 Hz, H-6″), 7.57–7.77 (m, 5H, ArH), 7.97–8.04 (m, 1H, ArH), 8.06–8.17 (m, 6H, ArH ), 8.22 (dd, 1H, *J =* 1.7, 8.6 Hz, ArH) 8.36 (s, 1H, H-2″), 8.59 (s, 1H, H-5), 8.63 (m, 1H, ArH); ^13^C NMR (50 MHz, DMSO-*d*_6_) δ: 107.5 (d, *J_C4″-F_* = 25.5 Hz), 113.0 (d), 113.6 (d), 114.8 (d, *J_C7″-F_* = 9.5 Hz), 115.7 (d), 123.5 (d), 123.6 (d), 125.7 (s), 126.6 (d), 126.8 (dx2), 127.0 (d), 127.6 (d, *J_C6″-F_* = 21.1 Hz), 128.6 (d), 128.9 (d), 130.0 (dx2), 131.2 (s), 132.8 (s), 133.7 (s), 134.9 (s), 135.0(d), 136.5 (s), 148.2 (s), 165.9 (s) 167.0 (s). Anal. Calcd. for C_27_H_17_FN_2_O_2_S_2_ C (66.92%) H (3.54%) N (5.78%) found C (66.73%) H (3.31%) N (5.64%).

##### 3-[2-(Naphthalen-2-yl)-1,3-thiazol-4-yl]-1-(phenylsulfonyl)-1*H*-indole (**4h**)

Conditions: reflux for 30 min; cycloexane/ethyl acetate 9:1;yellow solid; yield 94%; mp: 199–200 °C; IR: 1447, 1373 (SO_2_) cm^−1^; ^1^H NMR (200 MHz, DMSO-*d*_6_) δ: 7.41–7.52 (m, 2H, ArH), 7.57–7.75 (m, 5H, ArH), 7.98–8.17 (m, 6H, ArH), 8.23 (dd, 1H, *J =* 1.8, 8.6 Hz, ArH), 8.33 (s, 1H, H-2″), 8.39 (m, 1H, ArH ), 8.50 (s; 1H, H-5), 8.65 (m, 1H, ArH); ^13^C NMR (50 MHz, DMSO-*d*_6_) δ: 113.2 (s), 113.3 (d), 115.6 (d), 117.6 (s), 121.9 (d), 123.6 (d), 124.2 (d), 124.8 (d), 125.4 (d), 125.7 (d), 126.8 (dx2), 127.0 (d), 127.3 (d), 127.8 (d), 128.6 (d), 128.9 (d), 129.9 (dx2), 130.2 (s), 132.9 (s), 133.47 (s), 134.6 (s), 134.8 (d), 136.7 (s), 148.6 (s), 166.9 (s). Anal. Calcd. for C_27_H_18_N_2_O_2_S_2_ C (69.50%) H (3.89%) N (6.00%) found C (69.35%) H (3.98%) N (5.73%).

#### 3.1.6. Synthesis of 5-Substituted-3-[2-(naphthalen-2-yl)-1,3-thiazol-4-yl]-1*H*-indoles (**4i**–**l**)

To a suspension of the proper 3-[2-(naphthalen-2-yl)-1,3-thiazol-4-yl]-1-(phenylsulfonyl)-1*H*-indole **4e**–**h** (0.3 mmol) in ethanol (6.5 mL), a solution of sodium hydroxide (1.74 mmol, 0.07 g) in water (4.0 mL) was added. The reaction mixture was heated under reflux for 5–6 h. The solvent was evaporated under reduced pressure, and the resulting mixture neutralized with HCl 3N (2.0 mL) and extracted in ethyl acetate (3 × 50 mL). The organic phase was dried (Na_2_SO_4_), evaporated under reduced pressure and purified by column chromatography using cycloexane/ethyl acetate 7:3 as eluent.

##### 5-Methoxy-3-[4-(naphthalen-2-yl)-1,3-thiazol-2-yl]-1*H*-indole (**4i**)

Conditions: reflux for 6 h; yellow solid; yield 50%; mp: 175–176 °C; IR: 3378 (NH) cm^−1^; ^1^H NMR (200 MHz, DMSO-*d*_6_) δ: 3.90 (s, 3H, CH_3_), 6.85 (dd, 1H, *J =* 2.3, 8.8 Hz, H-6″), 7.39 (d, 1H, *J =* 8.8 Hz, H-7″), 7.58–7.63 (m, 2H, ArH), 7.72 (d, 1H, *J =* 2.3 Hz, H-4″), 7.89 (s, 1H, H-2″), 7.98–8.01 (m, 2H, ArH), 8.07–8.14 (m, 2H, ArH), 8.23 (dd, 1H, *J =* 1.5, 8.6 Hz, ArH), 8.62 (s, 1H, H-5), 11.34 (s, 1H, NH); ^13^C NMR (50 MHz, DMSO-*d*_6_) δ: 55.4 (q), 102.0 (d), 109.9 (d), 110.6 (s), 111.7 (d), 112.5 (d), 125.6 (s), 123.6 (d), 125.0 (d), 125.3 (d), 127.0 (d), 127.1 (d), 127.8 (d), 128.5 (d), 128.9 (d), 130.8 (s), 131.7 (s), 132.9 (s), 133.6 (s), 152.2 (s), 154.0 (s), 165.7 (s). Anal. Calcd. for C_22_H_16_N_2_OS C (74.13%) H (4.52%) N (7.86%) found C (73.88%) H (4.71%) N (8.03%).

##### 5-Bromo-3-[2-(naphthalen-2-yl)-1,3-thiazol-4-yl]-1*H*-indole (**4j**)

Conditions: reflux for 6 h; yellow solid; yield 68%; mp: 265–266 °C; IR: 3608 (NH) cm^−1^; ^1^H NMR (200 MHz, DMSO-*d*_6_) δ: 7.31 (dd, 1H, *J =* 1.9, 8.6 Hz, H-6″), 7.46–7.50 (m, 1H, H-7″), 7.59–7.64 (m, 2H, ArH), 7.95 (s, 1H, H-2″), 7.98–8.03 (m, 1H, ArH), 8.08–8.13 (m, 3H, ArH), 8.22 (dd, 1H, *J =* 1.8, 8.9 Hz, ArH), 8.40 (d, 1H, *J =* 1.8 Hz, ArH), 8.60 (s, 1H, H-5), 11.41 (s, 1H, NH); ^13^C NMR (50 MHz, DMSO-*d*_6_) δ: 110.8 (d), 110.9 (d), 122.3 (d), 123.5 (d), 124.12 (d), 125.4 (d), 126.9 (d), 127.0 (d), 127.2 (d), 127.8 (d), 128.5 (d), 128.9 (d), 130.7 (s), 132.9 (s), 133.6 (s), 135.5 (s), 141.8 (s), 151.4 (s), 154.2 (s), 161.6 (s), 166.0 (s). Anal. Calcd. for C_21_H_13_BrN_2_S C (62.23%) H (3.23%) N (6.91%) found C (62.48%) H (3.55%) N (6.75%)

##### 5-Fluoro-3-[4-(naphthalen-2-yl)-1,3-thiazol-2-yl]-1*H*-indole (**4k**)

Conditions: reflux for 5 h; white solid; yield 75%; mp: 227–228 °C; IR: 3124 (NH) cm^−1^; ^1^H NMR (200 MHz, DMSO-*d*_6_) δ: 7.06 (td, 1H, *J =* 2.5, 9.2 Hz, H-6″), 7.49 (dd, 1H*, J =* 4.7, 8.9 Hz, ArH), 7.60 (m, 2H, ArH), 7.95 (s, 1H, H-2″), 7.98–8.14 (m, 5H, ArH), 8.24 (dd, 1H, *J =* 1.7, 7.8 Hz, ArH), 8.92 (s, 1H, H-5), 11.62 (s, 1H, NH); ^13^C NMR (50 MHz, DMSO-*d*_6_) δ: 105.0 (d, *J_C4″-F_* = 24.3 Hz), 109.6 (d), 110.2 (d, *J_C6″-F_* = 24.3 Hz) 111.0 (s), 111.1 (s), 112.8 (d), 124.7 (s), 124.9 (s), 123.6 (d), 125.3 (d), 126.9 (s), 127.0 (d, *J_C7″-F_* = 3.3 Hz), (d), 127.2 (d), 127.8 (d), 128.6 (d), 128.8 (d), 130.7 (s), 132.9 (s), 133.3 (s), 151.6 (s), 166.0 (s). Anal. Calcd. for C_21_H_13_FN_2_S_2_ C (73.23%) H (3.80%) N (8.13%) found C (72.91%) H (4.05%) N (8.44%)

##### 3-[2-(Naphthalen-2-yl)-1,3-thiazol-4-yl]-1*H*-indole (**4l**)

Conditions: reflux for 5 h; light yellow solid; yield 80%; mp: 260–261 °C; IR: 2998 (NH) cm^−1^; ^1^H NMR (200 MHz, DMSO-*d*_6_) δ: 7.18–7.22 (m, 2H, ArH), 7.47–7.52 (m, 1H, ArH), 7.58–7.65 (m, 2H, ArH), 7.90 (s, 1H, H-2″), 7.98–8.16 (m, 4H, ArH), 8.21–8.28 (m, 2H, ArH), 8.62 (s, 1H, H-5), 11.50 (s, 1H, NH); ^13^C NMR (50 MHz, DMSO-*d*_6_) δ: 110.2 (d), 110.8 (s), 111.9 (d), 119.8 (d), 120.2 (d), 121.6 (d), 123.7 (d), 124.6 (s), 125.0 (d), 125.3 (d), 127.0 (d), 127.1 (d), 127.8 (d), 128.6 (d), 128.9 (d), 130.7 (s), 132.9 (s), 133.6 (s), 136.3 (s), 152.1 (s), 165.8 (s). Anal. Calcd. for C_21_H_14_N_2_S C (77.27%) H (4.32%) N (8.58%) found C (77.48%) H (4.25%) N (8.85%).

#### 3.1.7. Synthesis of 3-[2-(Naphthalen-2-yl)-1,3-thiazol-4-yl]-1*H*-pyrrolo[2,3-*b*]pyridines (**5a**–**d**)

To a suspension of naphthalene-2-carbothioamide **17** (0.07 g, 0.4 mmol) in anhydrous ethanol (15.0 mL), the proper 3-bromo-acetyl-pyrrolo[2,3-*b*]pyridine **15a**,**b** or **16a**,**b** (0.4 mmol) was added. The resulting mixture was heated under reflux for 5–6 h. After cooling, the precipitate formed was filtered off and recrystallized from ethanol.

##### 1-Methyl-3-[2-(naphthalen-2-yl)-1,3-thiazol-4-yl]-1*H*-pyrrolo[2,3-*b*]pyridine (**5a**)

Conditions: reflux for 5 h; yellow solid; yield: 75%; mp: 294–295 °C; ^1^H NMR (200 MHz, DMSO-*d_6_*) δ: 3.96 (s, 3H, CH_3_), 7.40 (dd, 1H, *J* = 5.0, 7.9 Hz, H-5″), 7.61 (m, 2H, ArH), 7.98–8.07 (m, 2H, H-2″, ArH), 8.11–8.15 (m, 2H, ArH), 8.22 (dd, 1H, *J* = 1.7, 8.6 Hz, ArH ), 8.32 (s, 1H, H-5), 8.46 (dd, 1H, *J* = 1.3, 5.0 Hz, H-6″), 8.63 (m, 1H, ArH), 8.84 (dd,1H, *J* = 1.3, 7.9 Hz, H-4″); ^13^C NMR (50 MHz, DMSO-*d_6_*) δ: 31.6 (q), 109.1 (s), 111.7 (d), 116.3 (d), 118.7 (s), 123.6 (d), 125.4 (d), 127.0 (d), 127.2 (d), 127.8 (d), 128.6 (d), 128.9 (d), 129.7 (d), 130.4 (s), 131.3 (d), 132.9 (s), 133.6 (s), 140.8 (d), 145.3 (s), 150.3 (s), 166.5 (s). Anal. Calcd per C_21_H_15_N_3_S: C (73.87%) H (4.43%) N (12.31%) found: C (73.62%) H (4.67%); N (12.60%).

##### 5-Bromo-1-methyl-3-[2-(naphthalen-2-yl)-1,3-thiazol-4-yl]-1*H*-pyrrolo[2,3-*b*]pyridine (**5b**)

Conditions: reflux for 4 h; yellow solid; yield: 55%; mp 252–253 °C; ^1^H NMR (200 MHz, DMSO-*d_6_*) δ: 3.90 (s, 3H, CH_3_), 7.58–8.63 (m, 2H, ArH), 7.96–8.01 (m, 1H, ArH), 8.05–8.12 (m, 3H, H-2″, ArH), 8.18 (dd, 1H, *J* = 1.7, 8.4 Hz, ArH), 8.28 (s, 1H, H-5), 8.42 (d, 1H, *J* = 2.1 Hz, ArH), 8.58–8.61 (m, 1H, ArH), 8.78 (d, 1H, *J* = 2.1 Hz, ArH); ^13^C NMR (50 MHz, DMSO-*d_6_*) δ: 31.2 (q), 108.2 (s), 111.7 (s), 111.8 (d), 118.8 (s), 123.5 (d), 125.5 (d), 127.0 (d), 127.2 (d), 127.7 (d), 128.5 (d), 128.9 (d), 130.4 (d), 130.5 (s), 130.6 (d), 132.9 (s), 133.6 (s), 143.1 (d), 146.0 (s), 150.0 (s), 166.4 (s). Anal. Calcd per C_21_H_14_BrN_3_S: C (60.01%) H (3.36%) N (10.00%) found: C (59.85%) H (3.60%) N (10.15%).

##### 3-[2-(Naphthalen-2-yl)-1,3-thiazol-4-yl]-1*H*-pyrrolo[2,3-*b*]pyridine (**5c**)

Conditions: reflux for 4 h; light brown solid; yield: 80%; mp: 277–278 °C; IR: 3126 (NH) cm^−1^; ^1^H NMR (200 MHz, DMSO-*d_6_*) δ: 7.35–7.43 (m, 1H, ArH), 7.59–7.65 (m, 2H, ArH), 7.99–8.04 (m, 1H, Ar), 8.07 (s, 1H, H-2″), 8.12–8.18 (m, 2H, H-5, ArH), 8.21–8.34 (m, 2H, ArH), 8.41–8.44(m, 1H, ArH), 8.63 (d, 1H, *J* = 9.5 Hz, ArH), 8.88 (t, 1H, *J* = 7.7 Hz, ArH), 12.40 (bs, 1H, NH); Anal. Calcd per C_20_H_13_N_3_S: C (73.37%) H (4.00%) N 12.83 found C (73.39%) H (4.11%) N (12.65%).

##### 5-Bromo-3-[2-(naphthalen-2-yl)-1,3-thiazol-4-yl]-1*H*-pyrrolo[2,3-*b*]pyridine (**5d**) 

Conditions: reflux for 4 h; white solid; yield: 85%; mp 300–301 °C; IR: 2906 (NH) cm^−1^; ^1^H NMR (200 MHz, DMSO-*d_6_*) δ: 7.62–7.67 (m, 2H, ArH), 7.58–8.03 (m, 1H, ArH), 8.09–8.13 (m, 3H, H-2″, ArH), 8.20–8.25 (m, 2H, H-5, ArH), 8.39 (bs, 1H, ArH), 8.62 (bs, 1H, Ar), 8.82 (bs, 1H, Ar), 12.30 (bs, 1H, NH). Anal. Calcd per C_20_H_12_BrN_3_S: C (59.12%) H (2.98%) N (10.34%) found C (59.29%) H (3.15%) N (10.71%).

#### 3.1.8. Synthesis of 3-[4-(Naphthalen-2-yl)-1,3-thiazol-2-yl]-1*H*-indoles (**6a**–**h**)

To a solution of the proper indolo-3-carbothioamide **22d**, **23a**–**d**, **24a**–**c** (0.91 mmol) in dimethylformamide (6.0 mL), naphthalene-2-acetylbromide **25** (0.23 g, 0.91 mmol) was added. The resulting reaction mixture was heated for 3–6 h at 60 °C or for 24 h at reflux. After reaction completion, monitored by TLC, water (12.0 mL) was added and the formed precipitate was filtered off. The crude obtained was then purified by column chromatography using cycloexane/ethyl acetate 7:3 as eluent.

##### 5-Methoxy-1-methyl-3-[4-(naphthalen-2-yl)-1,3-thiazol-2-yl]-1*H*-indole (**6a**)

Conditions: 60 °C for 6 h; brown solid; yield 98%; mp: 126–127 °C; cycloexane/ethyl acetate 95:5; ^1^H NMR (200 MHz, DMSO-*d*_6_) δ: 3.88 (s, 3H, CH_3_), 3.94 (s, 3H, CH_3_), 6.97 (dd, 1H, *J =* 2.5, 8.9 Hz, H-6″), 7.47–7.58 (m, 3H, ArH), 7.93–8.05 (m, 4H, ArH), 8.08 (s, 1H, H-2″), 8.18 (s, 1H, H-5), 8.22 (dd, 1H, *J =* 1.6, 8.6 Hz, ArH), 8.66 (s, 1H, ArH); ^13^C NMR (50 MHz, DMSO-*d*_6_) δ: 33.0 (q), 55.2 (q), 102.3 (d), 109.2 (s), 110.9 (d), 111.5 (d), 112.3(d), 124.3 (d), 124.6 (d), 125.2 (d), 126.1 (s), 126.6 (d), 127.6 (d), 128.2 (d), 128.3 (d), 130.9 (d), 131.9 (s), 132.2 (s), 132.6 (s), 133.2 (s), 153.7 (s), 155.0 (s), 162.7 (s). Anal. Calcd. for C_23_H_18_N_2_OS C (74.57%) H (4.90%) N (7.56%) found C (74.25%) H (5.15%) N (7.37%).

##### 5-Bromo-1-methyl-3-[4-(naphthalen-2-yl)-1,3-thiazol-2-yl]-1*H*-indole (**6b**)

Conditions: 60 °C for 6 h; cycloexane/ethyl acetate 9:1; orange solid; yield 98%; mp: 182–183 °C; ^1^H NMR (200 MHz, DMSO-*d*_6_) δ:3.90 (s, 3H, CH_3_), 7.45 (dd, 1H, *J =* 1.9, 8.8 Hz, H-6″), 7.51–7.61 (m, 3H, ArH), 7.95 (d, 1H, *J =* 2.8 Hz, ArH), 7.98–8.06 (m, 2H, ArH), 8.12 (s, 1H, H-2″), 8.20 (dd, 1H, *J =* 1.4, 8.6 Hz, ArH), 8.29 (s, 1H, H-5), 8.54 (d, 1H, *J =* 1.7 Hz, ArH), 8.61 (s, 1H, ArH); ^13^C NMR (50 MHz, DMSO-*d*_6_) δ: 33.1 (q), 109.0 (s), 111.6 (d), 112.9 (d), 113.9 (s), 122.7 (d), 124.3 (d), 124.6 (d), 125.0 (d), 126.1(s), 126.2 (d), 126.6 (d),127.7 (d), 128.1 (d), 128.4 (d), 131.7 (s), 132.1 (d), 132.6 (s), 133.1 (s), 135.8 (s), 154.0 (s), 162.0 (s). Anal. Calcd. for C_22_H_15_BrN_2_S C (63.01%) H (3.61%) N (6.68%) found C (63.36%) H (3.41%) N (6.85%).

##### 5-Fluoro-1-methyl-3-[4-(naphthalen-2-yl)-1,3-thiazol-2-yl]-1*H*-indole (**6c**)

Conditions: 60 °C for 6 h; cycloexane/ethyl acetate 8:2; orange solid; yield 75%; mp: 182–183 °C; ^1^H NMR (200 MHz, DMSO-*d*_6_) δ: 3.91 (s, 3H, CH_3_), 7.20 (dt, J = 2.6, 9.1, H-6″), 7.50–7.65 (m, 3H, ), 7.93–8.14 (m, 5H, H-2″, ArH), 8.22 (d, 1H, J = 1.7, 8.6 Hz, ArH), 8.31 (s, 1H, H-5), 8.63 (s, 1H, ArH); ^13^C NMR (50 MHz, DMSO-*d*_6_) δ: 105.4 (d, *J_C4″-F_* = 24.7 Hz), 109.5 (s), 110.7 (d, *J_C6″-F_* = 26.0 Hz), 112.0 (d), 111.0 (d), 112.1 (d), 124.3 (d), 124.3 (s), 124.6 (d), 126.1 (d), 126.5 (d), 127.6 (d), 128.3 (d, *J_C7″-F_* = 3.8 Hz), 131.7 (s), 132.6 (s), 133.2 (s), 133.4 (d), 133.8 (s), 154.0 (s), 162.3 (s) Anal. Calcd. for C_22_H_15_FN_2_S C (73.72%) H (4.22%) N (7.82%) found C (73.36%) H (4.45%) N (7.95%).

##### 1-Methyl-3-[4-(naphthalen-2-yl)-1,3-thiazol-2-yl]-1*H*-indole (**6d**)

Conditions: reflux for 24 h; cycloexane/ethyl acetate 9:1; orange solid; yield 99%; mp: 166–167 °C; ^1^H NMR (200 MHz, DMSO-*d*_6_) δ: 3.91 (s, 3H, CH_3_), 7.30–7.38 (m, 2H, ArH ), 7.53–7.61 (m, 3H, ArH), 7.93–8.08 (m, 3H, ArH ), 8.10 (s, 1H, H-2″), 8.20 (d, 1H, *J =* 1.4 Hz, ArH), 8.25 (s, 1H, H-5), 8.39–8.44 (m, 1H, ArH), 8.66 (s, 1H, ArH); ^13^C NMR (50 MHz, DMSO-*d*_6_) δ: 32.8 (q), 109.5 (s), 110.6 (d), 110.7 (s), 111.2 (d), 120.6 (d), 121.1 (d), 122.5 (d), 124.4 (d), 124.6 (d), 126.1 (d), 126.5 (d), 127.6 (d), 128.2 (d), 128.3 (d), 130.7 (d), 131.8 (s), 132.6 (s), 133.2 (s), 137.1 (s), 153.9 (s), 162.6 (s). Anal. Calcd per C_22_H_16_N_2_S: C (77.62%) H (4.74%) N (8.23%) found C (77.45%) H (4.39%) N (8.05%).

##### 5-Methoxy-3-[4-(naphthalen-2-yl)-1,3-thiazol-2-yl]-1*H*-indole (**6e**)

Conditions: 60 °C for 6 h; cycloexane/ethyl acetate 9:1; light brown solid; yield 48%; mp: 145–146 °C; IR: 3019 (NH) cm^−1^; ^1^H NMR (200 MHz, DMSO-*d*_6_) δ: 3.94 (s, 3H, CH*_3_*), 6.92 (dd, 1H, *J =* 2.5, 8.8 Hz, H-6″), 7.43 (d, 1H, *J =* 8.8 Hz, H-7″), 7.53–7.58 (m, 2H, ArH), 7.94 (d, 1H, *J =* 2.5 Hz, H-4″), 7.98–8.05 (m, 3H, H-2″, ArH), 8.08 (s, 1H, H-5), 8.15 (d, 1H, *J =* 2.8 Hz, ArH), 8.22 (dd, 1H, *J =* 1.5, 8.6 Hz, ArH), 8.66 (s, 1H, ArH), 11.70 (s, 1H; NH); ^13^C NMR (50 MHz, DMSO-*d*_6_) δ: 55.2 (q), 102.1 (d), 110.3 (s), 110.9 (d), 112.4 (d), 113.0 (d), 124.3 (d), 124.6 (d), 124.9 (s), 126.1 (d), 126.6 (d), 127.2 (d), 127.6 (d), 128.2 (d), 128.3 (d), 131.6 (s), 131.9 (s), 132.6 (s), 133.2 (s), 153.7 (s), 154.7 (s), 163.1 (s). Anal. Calcd. for C_22_H_16_N_2_OS C (74.13%) H (4.52%) N (7.86%) found C (74.44%) H (4.87%) N (7.66%).

##### 5-Bromo-3-[4-(naphthalen-2-yl)-1,3-thiazol-2-yl]-1*H*-indole (**6f**)

Conditions: 60 °C for 3 h; cycloexane/ethyl acetate 8:2; dark brown solid; yield 75%; mp: 224–225 °C; IR 3202 (NH) cm^−1^; ^1^H NMR (200 MHz, DMSO-*d*_6_) δ:7.40 (dd, 1H, *J =* 1.8, 8.7 Hz, H-6″), 7.49 (s, 1H, H-4″), 7.54–7.62 (m, 2H, H-7″, ArH), 7.94–8.06 (m, 3H, H-2″, ArH ), 8.12 (s, 1H, H-5), 8.21 (dd, 1H, *J =* 1.4, 8.6 Hz, ArH), 8.28 (d, 1H, *J =* 2.8 Hz, ArH), 8.55 (d, 1H, *J =* 1.4 Hz, ArH), 8.62 (s, 1H, ArH), 12.02 (s, 1H, NH); ^13^C NMR (50 MHz, DMSO-*d*_6_) δ: 110.1 (s), 111.6 (d), 113.4 (s), 114.3 (d), 122.6 (d), 124.3 (d), 124.6 (d), 125.1 (d), 126.0 (s), 126.2 (d), 126.6 (d), 127.7 (d), 128.2 (d), 128.3 (d), 128.4 (d), 131.8 (s), 132.6 (s), 133.2 (s), 135.3 (s), 153.9 (s), 162.4 (s). Anal. Calcd. for C_21_H_13_BrN_2_S C (62.23%) H (3.23%) N (6.91%) found C (62.38%) H (3.11%) N (7.23%).

##### 5-Fluoro-3-[4-(naphthalen-2-yl)-1,3-thiazol-2-yl]-1*H*-indole (**6g**)

Conditions: reflux for 24 h; cycloexane/ethyl acetate 7:3; brown solid; yield 60%; mp: 192–193 °C; IR 3205 (NH) cm^−1^; ^1^H NMR (200 MHz, DMSO-*d*_6_) δ: ^1^H NMR (200 MHz, DMSO-*d*6) δ: 7.13 (dt, 1H, *J =* 2.6, 9.2 Hz, H-6″), 7.51–7.61 (m, 3H, ArH), 7.93–8.13 (m, 5H, H-2″, H-5, ArH), 8.23 (dd, 1H, *J =* 1.7, 8.6 Hz, ArH), 8.28 (d, 1H, *J =* 2.9 Hz, ArH), 8.64 (s, 1H, ArH), 11.93 (s, 1H, NH); ^13^C NMR (50 MHz, DMSO-*d*_6_) δ: 105.2 (d, *J_C6″-F_* *=* 24.4 Hz), 110.5 (d), 110.7 (s), 111.3 (d), 113.3 (d), 113.5 (d), 113.6 (d), 124.4 (d), 124.6 (d), 124.7 (s), 126.2 (s), 126.3 (d, *J_C6″-F_* *=*18.5 Hz ), 127.6 (d), 128.3 (d, *J_C6″-F_ =* 3.7 Hz), 128.7 (d), 131.8 (s), 132.6 (s), 133.2 (s), 133.3 (s), 157.1 (s, *J_C5″-F_* *=*322.8 Hz), 162.7 (s). Anal. Calcd. for C_21_H_13_FN_2_S C (73.23%) H (3.80%) N (8.13%) found C (72.98%) H (4.17%) N (8.31%).

##### 3-[4-(Naphthalen-2-yl)-1,3-thiazol-2-yl]-1*H*-indole (**6h**)

Conditions: 60 °C for 24 h; cycloexane/ethyl acetate 9:1; orange solid; yield 60%; mp: 172–173 °C; IR 2972 (NH) cm^−1^; ^1^H NMR (200 MHz, DMSO-*d*_6_) δ: 7.29–7.35 (m, 2H, ArH), 7.55–7.62 (m, 3H, ArH), 7.97–8.13 (m, 3H, ArH), 8.14 (s, 1H, H-2″), 8.24–8.30 (m, 2H, H-5, ArH), 8.42–8.47 (m, 1H, ArH), 8.70 (s, 1H, ArH), 11.85 (s, 1H, NH); ^13^C NMR (50 MHz, DMSO-*d*_6_) δ: 110.6 (s), 111.2 (d), 112.2 (d), 120.2 (s), 120.4 (d), 120.9 (d), 122.4 (d), 124.3 (d), 124.6 (d), 126.1 (d), 126.5 (d), 126.8 (d), 127.6 (d), 128.2 (d), 128.3 (d), 131.8 (s), 132.6 (s), 133.2 (s), 136.6 (s), 153.9 (s), 163.0 (s). Anal. Calcd. for C_21_H_14_N_2_S C (77.27%) H (4.32%) N (8.58%) found C (77.55%) H (4.47%) N (8.65%).

### 3.2. Biology Studies

#### 3.2.1. Biology

HCT 116 cells (colorectal carcinoma), MDA-MB-435 cells (melanoma) and MCF-7 cells (breast cancer) were purchased from American Type Culture Collection, Rockville, MD, USA and grown in RPMI medium supplemented with L-glutamine (2 mM), 10% fetal bovine serum (FBS), penicillin (100 U/mL), streptomycin (100 μg/mL) and gentamicin (5 μg/mL). Cells were maintained in log phase by seeding twice a week at a density of 3 × 10^8^ cells/L in humidified 5% CO_2_ atmosphere, at 37 °C. In all experiments, cells were made quiescent through overnight incubation before the treatment with the compounds or vehicle alone (control cells) No differences were found between cells treated with DMSO 0.1% and untreated cells in terms of cell number and viability.

#### 3.2.2. Viability Assay In Vitro

Cytotoxic activity of the compounds against human tumor cell lines was determined by the MTT colorimetric assay based on the reduction of 3-(4,5-dimethyl-2-thiazolyl)bromide-2,5-diphenyl-2H-tetrazolium to purple formazan by mitochondrial dehydrogenases of living cells. This method is commonly used to illustrate inhibition of cellular proliferation. Monolayer cultures were treated with various concentrations (0.1–100 μM) of the drugs. Briefly, all cell lines were seeded at 2 × 10^4^ cells/well in 96-well plates containing 200 μL RPMI. When appropriated, cells were washed with fresh medium and incubated with the compounds in RPMI. After 72 h incubation, cells were washed, and 50 μL FBS-free medium containing 5 mg/mL MTT were added. The medium was discarded after 2 h incubation at 37 °C by centrifugation, and formazan blue formed in the cells was dissolved in DMSO. The absorbance, measured at 570 nm in a microplate reader (Bio-RAD, Hercules, CA, USA), of MTT formazan of control cells was taken as 100% of viability. The growth inhibition activity of compounds was defined as GI_50_ value which represents the log of the molar concentration of the compound that inhibits 50% cell growth. Each experiment was repeated at least three times in triplicate to obtain the mean values. 

#### 3.2.3. Measurement of Phosphatidylserine (PS) Exposure

The apoptosis-induced PS externalization to the cell surface was measured by flow cytometry by double staining with Annexin V-Fluorescein isothiocyanate (Annexin V-FITC)/propidium iodide (PI). Annexin V binding to phosphatidylserine is used to identify the earliest stage of apoptosis. PI, which does not enter cells with intact membranes, is used to distinguish between early apoptotic cells (Annexin V-FITC positive and PI negative), late apoptotic cells (Annexin V-FITC/PI-double positive) or necrotic cells (Annexin VFITC negative and PI positive). MCF-7 cells were treated with 3-[2-(naphthalen-2-yl)-1,3-thiazol-4-yl]-1*H*-indoles **4a**–**l** 3-[2-(naphthalen-2-yl)-1,3-thiazol-4-yl]-1*H*-pyrrolo[2,3-*b*]pyridines **5a**–**d**, and 3-[4-(naphthalene-2-yl)-1,3-thiazol-2-yl]-1*H*-indoles **5a**–**h**, prepared as described above. The compounds were dissolved in dimethyl sulfoxide (DMSO) and then diluted in culture medium to have a DMSO concentration not exceeding 0.1%. MCF-7 cells (5.0 × 10^4^ cells/cm^2^) were seeded in triplicate in 24-wells culture plates. After an overnight incubation, the cells were washed with fresh medium and incubated with the compounds or vehicle alone (control cells) in RPMI for 24 h. Then, cells were harvested by trypsinization and adjusted at 1.0 × 10^6^ cells/mL with combining buffer according to the manufacturer’ instructions (eBioscience, San Diego, CA, USA). One hundred μL of cell suspensions were added to a new tube, and incubated with Annexin V-FITC and PI solution at room temperature in the dark for 15 min. Then samples of at least 1.0 × 10^4^ cells were subjected to fluorescence-activated cell sorting (FACS) analysis by Epics XL™ flow cytometer using Expo32 software (Beckman Coulter, Fullerton, CA, USA) using appropriate bidimensional gating method.

#### 3.2.4. Cell Cycle Analysis

Cell cycle stage was analyzed by flow cytometry. MCF-7 cells (5.0 × 10^4^ cells/cm^2^) were seeded in triplicate in 24-wells culture plates. After an overnight incubation, the cells were washed with fresh medium and incubated with the compounds or vehicle alone (control cells) in RPMI for 24 h. Then cells were harvested by trypsinization. Aliquots of 1 × 10^6^ cells were washed with PBS and incubated in the dark in a PBS solution containing 20 μg/mL propidium iodide (PI) and 200 μg/mL RNase, for 30 min, at room temperature. Then samples of at least 1.0 × 10^4^ cells were subjected to FACS analysis.

## 4. Conclusions

New thiazole nortopsentin analogs in which one of the two indole units was replaced by a naphthalyl portion were conveniently synthesized. Among these, compounds **4a**, **6a** and **6d** showed good antiproliferative activity in particular against MCF7 cell line with GI_50_ values in the micromolar range. Biological studies performed to clarify their mechanism of action showed that the three compounds act as pro-apoptotic agents inducing a clear shift of viable cells towards early apoptosis in MCF-7 cells after 24 h treatment, while not exerting necrotic effects. They also caused cell cycle perturbation with significant decrease in the percentage of cells in the G0/G1 and S phases, accompanied by a concomitant percentage increase of cells in the G2/M phase, and appearance of a subG1-cell population.

## Figures and Tables

**Table 1:** 3-[2-(naphthalen-2-yl)-1,3-thiazol-4-yl]-1*H*-indoles **4** and 3-[2-(naphthalen-2-yl)-1,3-thiazol-4-yl]-1*H*-pyrrolo[2,3-*b*]pyridines **5**.

Compound	Substrate	R	R_1_	Y	Yields (%)
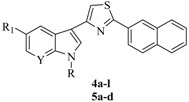					
**4a**	**11a**	Me	OMe	CH	95
**4b**	**11b**	Me	Br	CH	72
**4c**	**11c**	Me	F	CH	48
**4d**	**11d**	Me	H	CH	75
**4e**	**12a**	SO_2_Ph	OMe	CH	90
**4f**	**12b**	SO_2_Ph	Br	CH	70
**4g**	**12c**	SO_2_Ph	F	CH	60
**4h**	**12d**	SO_2_Ph	H	CH	94
**4i**	**4e**	H	OMe	CH	50
**4j**	**4f**	H	Br	CH	68
**4k**	**4g**	H	F	CH	75
**4l**	**4h**	H	H	CH	80
**5a**	**16a**	Me	H	N	75
**5b**	**16b**	Me	Br	N	55
**5c**	**15a**	H	H	N	80
**5d**	**15b**	H	Br	N	85

**Table 2:** 3-[4-(Naphthalene-2-yl)-1,3-thiazol-2-yl]-1*H*-indoles **6a**–**h**.

Compound	Substrate	R	R_1_	Yields (%)
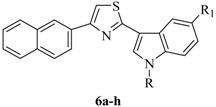				
**6a**	**23a**	Me	OMe	98
**6b**	**23b**	Me	Br	98
**6c**	**23c**	Me	F	75
**6d**	**23d**	Me	H	99
**6e**	**24a**	H	OMe	48
**6f**	**24b**	H	Br	75
**6g**	**24c**	H	F	60
**6h**	**22d**	H	H	60

**Table 3:** One dose (10^−5^ M) cytotoxic activity of compounds **4**–**6**.

Growth Percent ^1^			
Compound	HCT116	MCF-7	MDA-MB-435
**4a**	85.6 ± 4.3	24.9 ± 1.9	87.7 ± 4.1
**4c**	87.6 ± 5.2	74.5 ± 4.3	87.9 ± 5.3
**4i**	86.5 ± 4.8	84.8 ± 5.4	94.8 ± 5.5
**5b**	91.7 ± 5.4	62.9 ± 4.2	96.4 ± 4.9
**5d**	103.5 ± 2.3	47.1 ± 3.0	101.4 ± 3.2
**6a**	87.9 ± 3.8	30.7 ± 3.1	70.1 ± 4.2
**6c**	83.1 ± 4.1	50.3 ± 4.8	39.1 ± 2.7
**6d**	95.3 ± 4.5	37.5 ± 2.2	82.2 ± 5.1
**6g**	91.4 ± 6.4	41.6 ± 2.9	98.4 ± 4.6

^1^ Cells were treated with the compounds for 72 h and cell survival was measured by MTT assay in comparison to cells treated with vehicle alone (control), as reported in Section 3.2. Values are the mean ± SD of two separate experiments carried out in duplicate.

**Table 4:** GI_50_ values of the most active compounds **4a**, **6a** and **6d**.

Compound	GI_50_ (µM) ^1^
**4a**	2.13±0.12
**6a**	3.26±0.19
**6d**	5.14±0.34

^1^ Values were calculated using non-linear regression and are the mean ± SD of three separate experiments carried out in triplicate.
